# Does the Inverted Kidney Transplantation Technique Promote the Feasibility and Safety of Right Living Donor Nephrectomy?

**DOI:** 10.5152/tud.2022.22108

**Published:** 2022-07-01

**Authors:** Nasreldin Mohammed, Mohammed Ali Zarzour, Amr Mostafa Abdelgawad, Hamdy Mohammed Ibrahim, Paolo Fornara, Rabea Ahmed Gadelkareem

**Affiliations:** 1 Clinic for Urology and Kidney Transplantation Center, University Medical School of Martin-Luther-University Halle/Wittenberg, Germany; 2 Assiut Kidney Transplantation Center, Assiut Urology and Nephrology Hospital, Assiut University, Assiut, Egypt; 3 Department of Radiology, Faculty of Medicine, Assiut University, Assiut, Egypt

**Keywords:** Inverted kidney transplantation, kidney transplantation, laparoscopic donor nephrectomy, living donor nephrectomy, right donor nephrectomy

## Abstract

**Objective:**

The aim of this study is to compare the outcomes of right- and left-sided live donor nephrectomies using the inverted kidney transplantation technique for right live donor nephrectomy on transplantation.

**Material and methods:**

A retrospective review was done for the cases of live donor nephrectomy, either as open donor nephrectomy or laparoscopic donor nephrectomy between 2004 and 2019. Inverted kidney transplantation was used with right-sided grafts. The variables of the right- and left-sided live donor nephrectomies were compared.

**Results:**

There were 202 live donor nephrectomies including 71 (35.1%) open donor nephrectomies and 131 (64.9%) laparoscopic donor nephrectomies with 4 cases of conversion to open donor nephrectomy. There were 119 (58.9%) right-sided and 83 (41.1%) left-sided live donor nephrectomies with insignificantly different mean operative time (123 and 127 minutes; *P* = .09), mean warm ischemia time (82.3 and 84.5 seconds; *P* = .32), and mean blood loss (73 and 78 mL; *P* = .18), respectively. Inverted kidney transplantation was performed for 86% of grafts from right live donor nephrectomies. Discharge from hospital was on an average of 4.3 days postoperatively. There were only 3 complications (1 in right live donor nephrectomy and 2 in left live donor nephrectomies) with grade 2 according to Clavien–Dindo Classification. Incidence of delayed graft function (*P* = .09), transplant vein thrombosis (1 case in each group), 1-year graft survival rate (93.2% vs. 94.8%; *P* = .12), and 1-year serum creatinine levels (1.4 ± 0.3 vs. 1.3 ± 0.2; *P* = .09) revealed statistically insignificant differences.

**Conclusion:**

Regardless of the surgical technique, the right live donor nephrectomy seems to be technically as safe as the left live donor nephrectomy for both the donors and the recipients. Using inverted kidney transplantation provided convenient extensions of graft’s vessels to full length with no significant increased incidence of vascular thrombosis.

**Main Points**

- The right living donor nephrectomy is technically as safe as the left side procedure.
- The right living donor nephrectomy seemed to be safe and feasible for both the donors and the recipients, regardless of the surgical technique of nephrectomy.
- Both right open and laparoscopic donor nephrectomy result in convenient lengths of the right renal vessels without increased risks of vascular thrombosis.
- The inverted kidney transplantation technique seemed to be a good solution to overcome the shortness of right-sided graft vessels, avoiding the potential need for venous extenders.

## Introduction

Live donor kidney transplantation is widely recognized as an effective treatment for patients with end-stage renal disease.^1^ The clinical and social significance of living kidney donation has increased in recent years as the gap between needing and available donor organs has continuously been growing due to a decline in the number of deceased kidney donations.^1,2^ While living kidney donation represents the only source of grafts in many developing countries, its proportions in European countries have steadily progressed over the past decade.^2^ Nevertheless, the effectiveness and safety of surgery for live kidney donation are a major concern for both the donor and the recipient. As a result, optimization of surgical techniques and postoperative management of live donor nephrectomy (LiDN) remains important.^3^

Besides legislations, the general requirements for donor protection warrant keeping the better functioning kidney of the donor while donating a suitable one to the recipient.^4^ The method of live kidney donation has been further developed in parallel with the introduction of laparoscopic nephrectomy techniques.^2^ Due to the longer renal vessels, the left kidney remained the preferred side of LiDN. Despite this, there is still controversy over the absolute benefits of the left LiDN.^2^ Here, we present our long-term data from a comparison of right- and left-sided LiDN from 2 academic centers from different countries.

## Materials and Methods

From December 2004 to December 2019, a total of 202 LiDN, either as open donor nephrectomy (OpDN) or laparoscopic donor nephrectomy (LaDN), were performed in 2 academic centers, one in a European country and the other in a Middle-Eastern country. We followed the principles of the Declaration of Helsinki and its updates for conducting this study and it was approved by Assiut University Faculty of Medicine (17200148/2017). Written informed consent was obtained from the donors and recipients for participating in this study.

### Preoperative Workups

The preoperative donor workups were standardized in both academic institutions. Potential donors were screened by medical history, physical examination, and an array of laboratory tests, including urine analysis, viral studies, and immunologic studies that determine donor–recipient match. Also, chest and kidney imaging, including renal scintigraphy, was performed. Furthermore, all donors were evaluated by a clinical psychologist.

The decision to choose the side of the donated kidney was made mainly on the basis of the results of renal scintigraphy. Thus, the kidney with poorer function had to be donated strictly, when the difference between the split scintigraphy functions is ≥6 mL/min. Also, this procedure only deviated in 7 cases as the difference between the 2 kidneys was within <6 mL/min. This deviation was due to the presence of multiple renal vessels, where the potential for occlusion and perfusion failure was anticipated.

Outcome data of donors, including age, gender, body mass index (BMI), preoperative kidney scintigraphy, vascular studying, blood loss, intra- and perioperative complications, and graft outcome in recipients were collected retrospectively from both academic centers. In addition, literature from the last 10 years has been reviewed for similar results using the keywords “live kidney donation” and “right donor nephrectomy.”

## Surgical Techniques of the Right Donor Nephrectomy

After preparing the patients, both donor and recipient were admitted to the operating theaters. For the right LaDN, it was performed as a terminal hand-assisted graft retrieval LaDN. The surgeon’s hand is introduced to help retract the inferior vena cava to the left side, allowing more distance to clamp the vein at its maximal length.^5^ For the right OpDN, however, a conventional extraperitoneal approach via a flank incision was used.^3^ Immediately after removal of the kidney, perfusion using the histidine-tryptophan-ketoglutarate (HTK) solution (Custodiol^®^, Koehler, Alsbach-Haenlein, Germany) solution was carried out. The recipient was prepared simultaneously in the neighboring operating room. This resulted in a reduction of the cold ischemia time to less than 30 minutes. 

### Recipients

Kidney transplantation was performed using the standard technique of pre-peritoneal placement in the iliac fossa. Graft vessels were anastomosed as end-to-side fashion to the external iliac vessels.^3^ In all cases, no venous extenders were needed. Instead, certain surgical maneuvers were employed, including the contralateral placement of the graft (inverted kidney transplantation technique; IKT).^6^ Also, maximal length dissection of the external iliac vein to the confluence of the superficial circumflex iliac vein, hand assistance in LaDN, and ligation of the internal iliac vein to render the external iliac vein flail in recipients were carried out. The immunosuppression protocol was standardized in all recipients consisting of a triple combination (tacrolimus, methylprednisolone, and mycophenolate mofetil). Patients with a particular immunological risk received an additional therapy with anti-thymocyte globulin or IL-2R inhibitor (basiliximab) as induction therapy. Perioperatively and during the first year postoperatively, we recorded survival rates of recipients and grafts, acute rejection rates, vein thrombosis, and ureteral complications, including the need for a percutaneous nephrostomy, ureter reconstructions, and renal function. The first-line management of acute rejection was the pulse doses of methyl-prednisolone, while the second line was anti-thymocyte globulin. Delayed graft function was defined as the need for dialysis within the first postoperative week. Complications were reported, according to the modified Clavien–Dindo Classification (CDC).

### Statistical Analysis

The statistical package for social sciences, version 20.0 (IBM SPSS Corp.; Armonk, NY, USA) was used for statistical analyses. Chi-square test and Student’s *t*-test were used for statistical comparisons. The rate of 1-year graft survival was calculated by Kaplan–Meier analysis. The level of significance was set at *P* < .05.

## Results

Characteristics, surgical and postoperative outcomes of donors, and those of recipients are shown in Tables 1 and 2. 

## Results in Donors

Overall, right- and left-sided LiDNs were performed in 119 (58.9%) and 83 (41.1%) donors, respectively. There were 121 female donors and 81 male donors with an average BMI of 26.17 kg/m^2^, an average intraoperative blood loss of 67.43 mL, a mean surgery time of 162.39 minutes, and mean warm ischemia of 81.69 seconds. The average scintigraphic function of the kidneys removed was 48.5%. In these results, there was no significant difference between right and left LiDNs (Table 1).

Regarding the settings, 149 (73.8%) and 53 (26.2%) LiDNs were performed in the European center and Middle Eastern center including 128 (85.9%) and 3 (5.7%) LaDNs, respectively. In LaDN group, a total of 4 conversions to OpDN occurred (2 for right LiDN), 3 of which were elective (CDC3b) and 1 was an emergency conversion (CDC4). An elective right-sided conversion was carried out because the bleeding situation from the detached vessels was unclear and 2 conversions were carried out for a left-sided LiDN—1 of them due to an unclear bleeding situation from the vascular stumps and 1 due to the very adherent perinephric fat. The emergency conversion took place in the context of a right LaDN during the retrocaval dissection of the renal artery with stapler dysfunction and arterial bleeding. This resulted in a massive hemorrhagic shock, from which the donor recovered without consequences. Overall, a total of 3 CDC2 complications occurred, namely, 2 postoperative urinary tract infections and 1 acute pancreatitis after left LiDN, which was managed conservatively.

When we compared the clinical results of right- to those of left-sided LiDN, no significant difference in intra- or postoperative results and complications was found. Mean operative time was 123 minutes (range, 102-182 minutes) in the right LiDN group and 127 minutes (range, 109-189 minutes) in the left LiDN group (*P* = .09). The warm ischemia time was not significantly different between the right LiDN (82.3 seconds; range, 13-280 seconds) and left LiDN (84.5 seconds; range, 12-301 seconds; *P* = .32). Also, the average estimated blood loss for the right LiDN (73 mL; range, 50-280 mL) was not significantly different from that of the left LiDN (78 mL; range, 49-320; *P* = .28). Blood transfusion was intraoperatively required for 1 donor of right LiDN and for 1 donor of left LiDN 2 days after donation. The mean time to discharge from the hospital was equal for the patients in both groups (4.2 days for right LiDN vs. 4.6 days for left LiDN; *P* = .73). 

## Results in Recipients

A total of 108 (53.5%) kidneys were transplanted to the left iliac fossa and 94 (46.5%) into the right fossa. Of the 119 right-sided grafts, IKT was done in 102 (85.7%) cases. The remaining 17 (14.3%) cases were transplanted to the right iliac fossa with maximal dissection of the external iliac vein and scarification of the internal iliac vein in 12 (18.1%) cases. 

The characteristics of the recipients after right- and left-sided LiDN were similar regarding age, sex, BMI, previous abdominal operations, second kidney transplantation, preemptive transplantation, dialysis time, panel reactive antibody test, and ischemia times. One out of 83 of the kidneys donated from the left side had venous thrombosis and 1/119 of those kidneys from the right side. Also, when comparing the rate of early function, no significant difference was observed between the recipients of both groups. Overall, 5 patients had delayed graft function, three in the left group and two in the right group. The 1-year graft survival rate was 93.2% in the right group versus 94.8% in the left group (*P *= .12). Further, serum parameters of the glomerular filtration rate for the characterization of renal function showed no statistically significant difference between the groups 1 year after transplantation.

## Discussion

The major drawbacks of using the right kidney for living kidney donation are the shorter length of the renal vein and the partly retrocaval position of the right renal artery.^6^ In addition, transection of the renal vessels with vascular staplers, as used in laparoscopic procedures, could lead to additional loss of available length required for implantation. Short vessels can consume more time and extend the length of the cold ischemia during renal graft vessel anastomosis. Thus, when reviewing the cumulative experience of LiDN, it is clear that the left kidney is preferred because of the longer renal vein. On the other side, drawbacks of the left LaDN are higher chances of lacerating the spleen during mobilization of the splenic flexure of the colon and vascular risks of handling the lumbar and adrenal tributaries of the left renal vein.^7^ The short right renal vein may generate surgical difficulties represented by challenges of performing sound vascular anastomoses. However, regardless of these potential difficulties, the principle of kidney donation and retrieval is that the best kidney should always remain with the donor.^4^

Both the current results and literature advocate the feasibility and safety of the right LiDN, for both the donor and the recipient. In reviewing the relevant literature, the period 2009-2019 was selected to search the PubMed database. The search revealed 12 relevant papers; our own papers were not included. Most of the publications identified were single-center publications (8/12), and only 4 multi-center studies or meta-analyses were available. All publications in the selected period advocate a general concept that the right LiDN is a safe procedure (Table 3). Particular attention should be paid to the work of Khalil et al^8^ published in 2016. The data from 58 599 donor nephrectomies from the Organ Procurement and Transplantation Network/United Network for Organ Sharing database were evaluated, of which, 8116 and 50 483 cases were right and left LiDNs, respectively. Overall, Khalil et al^8^ reported that the main difference in LiDN relates to the distinction between “high volume” and “low volume” centers. The high-volume centers usually perform LaDN, even with multiple vessels, and they have more right LiDNs. However, they have less complication rates, such as those of graft venous thrombosis. On the other hand, the low volume centers perform left OpDN more than LaDN, but they may have higher graft vein thrombosis with right LiDNs. The result of this database analysis was that the right LiDN is a safe procedure with comparable overall results, despite a slightly increased risk of graft vein thrombosis. In addition, it showed significantly higher rates of delayed graft function but without a worse overall outcome. However, 7-8 times more LiDNs have been performed on the left than on the right.^8^

Ravaioli et al^9^ evaluated a 10-year experience at Italian transplant centers and came to similar results. In this study, the rates of use of minimally invasive methods and complications were mainly dependent on the experience of the surgeon. Furthermore, other studies and meta-analyses by Liu et al^10^ and Broudeur et al^11^ also reported that right and left LiDN are comparable procedures with similar donor, recipient, and transplant safety and complication rates. Specifically, the study by Liu et al^10^ reported that the surgeon preference may affect accepting donors that are not compatible with the anatomical and functional criteria of donation. The results of many single-center studies are in concordance with the above-mentioned multi-center studies and literature analyses.^12-19^ By comparing the results of the current study with those from the literature, the results from both territories are correspondent so far. It is noticeable, however, that more right LiDNs are performed at our transplant centers than the average in the literature. Whether this is due to the fact that in our centers a decision is made strictly according to the functional share and an accumulation in favor of the right kidney at our centers is coincidental or is incomprehensible due to the prevalence in favor of the left kidney in other centers.

The current results may augment the evidence for the feasibility and safety of right LiDN relative to the left LiDN, when performed via both LaDN and OpDN techniques. In LaDN, the hand-assisted technique allowed the surgeon to get full-length right renal vein.^5^ Also, the long-term familiarity with OpDN might be the cause of having a surgical ability to retrieve right-sided kidney grafts with sufficient lengths of vessels. In the recipient surgery, we practiced 2 surgical procedures to enhance the feasibility of the right renal grafts. Dissection and scarification of the internal iliac vein enhanced the safety of the anastomosis by rendering the external iliac vein flail and mobile when anastomosed to the grafted vein. Also, IKT was another surgical procedure that enhanced the feasibility and safety of the anastomosis by bringing the graft vessels posterior near to the iliac vein. Similar to the current results, the IKT has been described in the literature as a favorable factor in enhancing the safety of the right LiDN technique.^6^

In conclusion, the current study revealed that regardless of the surgical technique, the right LiDN is technically as safe as the left LiDN. Both right OpDN and LaDN result in a convenient extension of right renal vessels without significantly increased risks of vascular thrombosis. Also, right LiDN seemed to be safe and feasible for both the donors and the recipients, regardless of the surgical technique of LiDN. In addition to the technical ability to retrieve full-length right-sided graft vessels, IKT and scarification of the internal iliac vein may provide favorable effects on avoiding the need for venous extenders and vascular complications. 

## Figures and Tables

**Table 1.** Demographic and Operative Characteristics of the Study Population: Right-Sided Versus Left-Sided Donor Nephrectomy

Characteristics	Right LiDN (n = 119)	Left LiDN (n = 83)	*P*
Age (years)			.43
Mean ± SD	51.3 ± 16.8	52.5 ± 18.1	
Range	20-72	21-76	
			
Female/male ratio	1.47	1.59	.19
Body mass index (kg/m^2^)	26.5	25.4	.18
ASA	1.4	1.5	.23
Operative time			
Median	123 minutes	127 minutes	.09
Range	102-182 minutes	109-189 minutes	
Warm ischemia time			
Mean	82.3 seconds	84.5 seconds	.32
Range	14-280 seconds	13-301 seconds	
Artery length (cm)	3.3	3.2	.76
Vein length (cm)	2.6	3.8	.04
Renal scintigraphy of donor kidney			.23
Mean	47.4%	48.2%	
Range	40%-52%	43%-56%	
Hospitalization period (mean)	4.2 days	4.6 days	.73
Operative blood loss			
Mean	73 mL	78 mL	.18
Range	50-280 mL	49-320 mL	
Intraoperative complications			.06
CDC-1	0	0	
CDC-2	1	2	
CDC-3a	0	0	
CDC-3b	1	2	
CDC-4	1	0	
CDC-5	0	0	

ASA, American Society of Anesthesiologists; LiDN, living donor nephrectomy; NS, not significant; SD, standard deviation; CDC, Clavien–Dindo Classification.

**Table 2.** Characteristics and Outcomes of Recipients of Right-Sided Versus Left-Sided Donor Nephrectomy

Charatcteristics	Right LiDN (n = 94)	Left LiDN (n = 108)	*P*
Age (years) Mean ± SDRange	48.4 ± 18.832-63	48.1 ± 19.134-62	.53
Female/male ratio	1.48	1.39	.23
Body mass index (kg/m^2^)	26.9	27.3	.24
PRA (%)	18.3	17.8	.21
Duration of dialysis (years) Mean Range	1.5 0-5.9	1.6 0-6.1	.34
Venous thrombosis (n)	1	1	NA
ABOi (n)	11	13	.73
Recipient diuresis (mean)			
POD1	5753 mL	5276 mL	.09
POD3	4423 mL	4213 mL	.09
POD7	3326 mL	3245 mL	.11
Graft function			
Delayed graft function	2 (2.1%)	3 (2.8%)	.09
One-year posttransplant graft survival	93.2%	94.8%	.12
Biochemical marker of GFR			
Mean serum creatinine at POD1 (mg/dL)	5.1	4.9	.14
Creatinine (mean ± SD) 1 year post-transplant	1.4 ± 0.3	1.3 ± 0.2	.09
Cystatine C (mg/L; mean ± SD)1 year post-transplant	1.7 ± 0.6	1.9 ± 0.6	.08

LiDN, living donor nephrectomy; SD, standard deviation; POD, postoperative day; PRA, panel reactive antibody; ABOi, ABO-incompatible; GFR, glomerular filtration rate.

**Table 3.** Publications in the Period of 2009-2019 Agreeing with the Safety of the Right Donor Nephrectomy Procedure

Author	Year of Publication	Type of Study	Number of Patients			Complications in Donors			Graft Vein Thrombosis		
All	RDN	LDN	RDN	LDN	*P*	RDN	LDN	*P*
Khalil et al^8^	2016	MC	58.599	8116	50.483	404	3446	ns	96	403	.006
Liu et al^10^	2013	MC	32.426	3566	28.860	1403	3664	ns	1053	432	.04
Broudeur et al^11^	2019	MC	-	2930	-	-	-	-	1	0	ns
Yoon et al^12^	2014	SC	2157	435	1722	-	-	-	-	-	-
Kumar et al^13^	2018	SC	1850	168	1682	4	6	-	-	-	-
Ravaioli et al^9^	2017	MC	693	159	534	3	5	-	3	2	ns
Qiu et al^14^	2017	SC	527	104	423	-	-	-	0	1	ns
Kashiwadate et al^15^	2015	SC	151	87	64	6	2	-	0	0	ns
Bagul et al^16^	2012	SC	303	59	244	-	-	-	0	0	-
Omoto et al^17^	2013	SC	533	24	509	4	33	ns	0	0	-
Bachir et al^18^	2011	SC	94	20	74	0	0	-	0	0	-
Tsoulfas et al^19^	2012	SC	279	19	260	2	19	-	-	-	-

MC, multi-center; SC, single-center; RDN, right donor nephrectomy; LDN, left donor nephrectomy; ns, not significant.
